# How online discussion board activity affects stock trading: the case of GameStop

**DOI:** 10.1007/s11408-022-00407-w

**Published:** 2022-03-30

**Authors:** André Betzer, Jan Philipp Harries

**Affiliations:** University of Wuppertal, Gaußstraße 20, 42119 Wuppertal, Germany

**Keywords:** GameStop, Retail trading, Trade volume, Market structure, D91, G14, G41

## Abstract

In January 2021, the stock price of NASDAQ-listed GameStop Corporation surged more than twenty-fold for no discernible economic reason. Many observers attributed this broadly covered rise to retail investors, organizing themselves in Reddit’s WallStreetBets community. While Social Media-organized trading is not a new phenomenon, the magnitude of the resulting swings in the share price and surge in trading volume of GameStop is unprecedented. Using financial data, as well as an extensive dataset of Reddit posts, we provide empirical evidence for the relationship of Reddit posts and GameStop (retail) trading. While we find a significant and positive relationship between Reddit posts and various trading measures in the following 30-min window in accordance with an attention-based mechanism, our results offer no indication for the informativeness of Reddit posts and hint at a complex and probably nonlinear interdependence between Social-media and trading activity, preventing proof of a one-directional, causal effect.

**Supplementary Information:**

The online version contains supplementary material available at 10.1007/s11408-022-00407-w.

## Introduction

Retail trading has soared since the beginning of 2020, stimulated by stay-at-home-advisory mandates and lack of other entertainment options on the one hand and new, accessible app-based brokers without order fees on the other hand. Citing data from web data provider SimilarWeb, Ozik et al. (2021) report a 115% increase in traffic in Q2/2020 for Robinhood, the most popular app-based retail broker along with large increases for other retail brokers. Investment firm Citadel estimates that retail traders more than doubled their share of stock-market activity in H1 2020 compared to 2019, accounting for more than 20% of trade volume with an even sharper rise of retail activity in the option market^1^ At the same time, sharing investment opinions in online communities has become a common phenomenon in recent years. While earlier social-media platforms with a focus on investments or stock markets mostly appealed to relatively few, sophisticated users and investment-specific data from general social-media platforms like Twitter or Facebook is sparse and difficult to filter, the WallStreetBets community on Reddit rose to almost 10 million users in early 2021 and contains more than 40 million user posts in our sample from the start of 2020 to March 2021, enabling us to analyze the relationship between posts and trading activity in 30-minute windows.

Among these posts, more than 4 million can be attributed to a single company whose share price rose more than 20-fold in January 2021 without any fundamental news release or discernible economic reason: GameStop. While several other factors, which are partially discussed in Sect. 3.1, may have contributed to the surge or could have ignited the initial interest in this specific company, media reports and online discussion suggest that WallStreetBets played a crucial role in this situation. The chair of the SEC, Gary Gensler, acknowledged that “this winter’s events also highlighted the rapidly changing face of social media and its intersection with our capital markets.”^2^ However, the nature of the relationship between social media posts and trading activity is less clear than it seems and data availability and noisiness problems are complicating the scientific and forensic examination of events. As high-frequency empirical data about how modern social media platforms affect trading activity is still scarce, the recent situation around GameStop is uniquely suited to analyze whether prior results can be transferred to this new high-volume social media platform in a stock market environment with increased volatility and retail participation.

While there is a breadth of literature showing that online activity can have an impact on the stock market (e.g., Antweiler and Frank 2004, with one of the first studies of a large social media dataset, showing a positive relationship between daily Yahoo message board comments and trading volume), the mechanism behind this relationship is still a much-discussed topic. Alternative explanations about how Internet postings affect individual’s decision-making include i) effects of general sentiment and disagreement (e.g., Tetlock 2007; García 2013; Cookson and Niessner 2020; Guégan and Renault 2021), ii) more specific social interaction and social transmission bias (e.g., Hirshleifer 2020; Cookson et al. 2021) or iii) the effect of investor attention itself, independent of the impact on beliefs and sentiment (e.g., Barber and Odean 2008; Da et al. 2011). Multiple recent working papers try to shed light on different aspects of the GameStop surge and disentangle possible effects (e.g., Long et al. (2021) and Umar et al. (2021) with a focus on sentiment; Hasso et al. (2021) and Pedersen (2021) on investor types and social interaction or Vasileiou et al. (2021) on web searches and attention); however empirical evidence of an intraday effect of Reddit comments on trading activity is still lacking.

Our contribution to the existing literature is twofold: First, our study centers on the question whether social media posts are indeed a short-term driver of (retail) stock trading activity and we can confirm previously observed relationships in a dynamic, highly unusual market environment. We also extend the existing evidence by adding option-based volume measures to our analyses, which, to the best of our knowledge, hasn’t been done before in this context as retail option trading is a fairly recent phenomenon. Second, we try to disentangle the effect on retail and on other trades and examine the informativeness of Reddit posts for future price changes. The informativeness of online posts and retail trading is still a controversial topic among researchers with conflicting results in different settings. Using the raw number of comments without regard for their content or tone, we add to the attention-based strand of literature on the relationship between social media and trading activity. Regardless the direction and exploitability of trading activity and pricing changes, the existence of a direct link between social media activity and stock buying is at odds with conventional economic theory and the efficient market hypothesis. This is especially apparent in the case of GameStop, where there was no confounding dissemination of material new information and a specific group of investors bought stocks and options at valuation levels, which far exceed any reasonable fair value. Our results show that an increase in Reddit posts on GameStop is followed by a significant increase in the GameStop trading volume for both, stocks and options in the following 30-minute window. This effect is economically large, robust over multiple specifications and found for both, the overall volume and volume of retail investors. However, we are not able to reliably discern between the effect on retail and other trades using multiple criteria to classify retail trades. Additionally, we cannot establish causality beyond reasonable doubt, as the effect is not one-directional and the logarithmic rise of comments and trade activity in the sample poses challenges for empirical analyses.

In further results, we don’t find that Reddit posts are informative for GameStop returns. There is no significant relationship between Reddit posts and realized abnormal returns in the following 30-min window. We also find no significant relationship to marketable retail order imbalances as defined by Boehmer et al. (2021). This confirms e.g., the results by Antweiler and Frank (2004) and validates the result of Ammann and Schaub (2021), that “mainly followers who are typically considered to be unsophisticated [...] trade after comment postings” on a recent, large-sample case study.

The remainder of this paper is organized as follows: Sect. 2 contains a brief literature review, first elaborating on possible different mechanisms explaining the impact of online activity on stock and option trading in Sect. 2.1 before reviewing previous results in Sect. 2.2. Section 3 introduces the institutional setting that makes our analysis relevant, with an overview of Reddit and WallStreetBets as a specialized but widely used social media platform in Sect. 3.1 and a perspective on the enormous growth of Retail trading in 2020 and 2021 in Sect. 3.2. Section 4 then explains data and methodology, before empirical results are presented and discussed in Sect. 5. Finally, Sect. 6 provides some directions for future research and concludes with a summary.

## The effect of social media posts on trading activity and returns

The possible effect of online activity on asset prices and whether social media activity can contain information for the formation of prices is the topic of many studies in the area after De Long et al. (1989) coined the term “noise trading” and Daniel et al. (2002) established that psychological biases affect investor behavior and prices and concluded that “if investors are foolishly aggressive in their trading, they may earn higher rewards for [...] exploiting information signals more aggressively.” Following them, Antweiler and Frank (2004) then showed empirically that a relationship between online activity and trading volume can be demonstrated, using a dataset of Yahoo! Finance and Raging Bull comments. Since then, the mechanisms through which online communities impact (retail) investor behavior are an important topic in economic research and are well-discussed.

### Possible explanations for a relationship between online and trading activity

The suggested mechanisms that affect the relationship of social media activity and trading activity by altering the decision-making of individual market participants can mainly be summarized into three strands of literature:

First, approaches that focus on investor *Sentiment*, including the tone, heterogeneous beliefs and also disagreement. Historically most research focused on price effects of indirectly measured, “top-down” sentiment (e.g., Baker and Wurgler 2007; Tetlock 2007; García 2013; Kumar et al. 2020, finding for example that tone in newspaper articles can lead to pricing anomalies and results that are “are consistent with theoretical models of noise and liquidity traders”). Related to our case study, Long et al. (2021) classify Reddit posts into sentiment categories and find that “both tone and number of comments influence GameStop intraday returns.” However, these effects are elusive and data suggests that textual sentiment classification into emotion-based categories is very challenging for WallStreetBets posts, as these contain a lot of different slang, memes and emoticons which are barely understandable for uninitiated readers or parsers.^3^ More recent research also focuses on “bottom-up” investor sentiment, demonstrating how individual sentiment can affect trading activity (e.g., Cookson and Niessner 2020, who show that disagreement on an online platform can have an impact on trading volume).

Second and related to the “bottom-up” sentiment approach, there is the recent strand of *Social Interaction*, going beyond the cursory and visible measures often used for sentiment analysis. For example, Heimer (2016) explains the disposition effect with social interaction and peer pressure. Hirshleifer (2020) introduced the term “social transmission bias” that offers an endogenous social explanation for “action booms, price bubbles, and swings in investor sentiment” in contrast to the exogenous explanation of most sentiment literature. One recent example is the “echo chambers” on the StockTwits social media platform analyzed by Cookson et al. (2021). We think that the existence of similar echo chambers on WallStreetBets is likely, but impossible to empirically validate in our GameStop case study.

Thus, we concentrate on the third explanation for the relationship of social media and trading activity: *Investor Attention*. Barber and Odean (2008) showed that individual investors are more likely to buy attention-grabbing stocks, employing a range of proxies like exceptional volume, returns or news coverage. Da et al. (2011) extended this approach using the Google search volume index. They argue that an impact on investor beliefs or sentiment is not necessary to drive retail trading volume. Ben-Rephael et al. (2017) further validate this hypothesis, showing a similar effect for institutional investors as well. More recently, Ammann and Schaub (2021) find empirical evidence for a significant correlation between public Internet postings by traders and the investment activity of followers. However, they also show, “that it is mainly followers who are typically considered to be unsophisticated who trade after comment postings.” Related to our empirical choices and concentration of raw post count as measure of attention, Behrendt and Schmidt (2018) find that the amount of social media posts yields better results compared to sentiment measures, using a Twitter dataset, fitting to our conjecture of an attention-based mechanism.

### Prior results on retail and online activity-induced trading and returns

While many studies find some significant relationship between online activity and trading volume, published results about the impact of investor attention and sentiment on future returns and the informativeness of online activity-induced trading are not very consistent. Whereas some published studies, at least partially, document a significant relation between online posts and returns (e.g., Chen et al. 2014; Avery et al. 2016; Crawford et al. 2017; Bradley et al. 2021, , the latter on a small subset of Reddit WallStreetBets posts), other researchers cannot confirm a significant relationship in that regard (e.g., Tumarkin and Whitelaw 2001; Dewally 2003; Antweiler and Frank 2004; Kim and Kim 2014; Behrendt and Schmidt 2018; Nisar and Yeung 2018; Ammann and Schaub 2021), which mirrors the results of studies surveying the effect of media news on stock returns (e.g., Campbell et al. 2012; García 2013; Ahmad et al. 2016).

With regards to retail trading in general, the empirical evidence on the predictability of future stock returns is mixed as well. While many early studies in this strand of literature such as Barber et al. (2006) find that individual investors trading provide no information for equity markets and prices and individual investors often achieve negative returns (e.g., Barber and Odean 2000), more recent studies such as Kelley and Tetlock (2013), Barrot et al. (2016) or Boehmer et al. (2021) find that retail investor trading can be informative and/or predict the cross section of future stock returns.

Fittingly for the GameStop scenario, Han and Kumar (2013) find empirical evidence that retail investors in contrast to institutional investors prefer “stocks with high volatility, high skewness and low prices.” In addition, the authors document that retail traders that prefer lottery stocks are often younger and male and have a lower income and lower education compared to other investors, as well as a strong gambling propensity (e.g., Kumar and Lee 2006, come to a similar conclusion), which matches well with commonly observable behavior of WallStreetBets users and could be an additional explanation for the lack of informativeness of comments.

## Institutional setting: WallStreetBets, GameStop and the surge of retail trading

### WallStreetBets and GameStop

Reddit is a social media platform that was founded in 2005. Like other social media platforms, contributors are able to post content which can then be commented on by other users. Reddit is a collection of forums, which are called subreddits and where each subreddit is dedicated to a specific topic. WallStreetBets, which is now one of Reddit’s largest subreddits with more than 11 million subscribers, was created in 2012 and focuses on speculative equity trading.^4^ As speculative trading and “gambling” is emphasized, it is reasonable to assume that retail trading activity originating from WallStreetBets may exhibit different characteristics than retail trading activity from more conventional sources of investment advice or discussion.

However, due to this explicit focus on speculative trading, the high probability that active WallStreetBets users indeed engage in stock and option trading^5^ and the high volume of posts that often concentrates on a few feverishly discussed stocks, WallStreetBets recently became a valuable data source for the analysis of retail investor behavior (see e.g., Long et al. 2021; Bradley et al. 2021) and we regard the platform as uniquely suited for our research.

Starting in 2020, users of the message board WallStreetBets on Reddit turned their eye on the stock of struggling video game retail company GameStop. While only a few users discussed the stock at first^6^, hundreds and thousands of retail investors joined them in early 2021, when the GameStop stock surged due to the expectation of an imminent short squeeze. While GameStop opened in 2021 on January 4th with a price of $19.03, the closing price on January 21th was already $43.03, an increase of more than 100%, without any new fundamental information released by the company in the meantime. However, the real surge had barely started by then: In the following five trading days, the share price increased 10-fold again and reached a top of $483 in the morning of Thursday, January 28th, before major brokers disabled market participants ability to open new or increase existing positions in GameStop. Huge losses of GameStop-shorting hedge funds, margin calls of unprecedented size and the failure of established risk management models led the CEO of Interactive Brokers, one of the biggest American brokers to the statement that “we have come dangerously close to the collapse of the entire system and the public seems to be completely unaware of that, including Congress and the regulators”^7^.

### Surge of retail trading in 2020 and 2021

A reason for the relevance of our contribution despite the large amount of existing literature on the relationship between social media activity and stock trading is the strong increase in retail trading that started in 2020 and which is partially attributable to the COVID-19 pandemic, which limited many day-to-day (out-of-home) activities for a big part of 2020 and 2021.

Analysts from Credit Suisse estimated in February 2021 that retail trading as a share of overall market activity had nearly doubled from between 15% and 18% to over 30% since the start of 2020.^8^ An additional reason for increased retail trading has been the decision of many retail-focused US brokers to drop trading fees in the fall of 2019. Robinhood, an app-based broker with more than 3 million app downloads in January 2021^9^, is the most notable of a new kind of brokers which gamify trades and make stock and option trading available to a new demographic that also exhibits high social media affinity and activity. Ozik et al. (2021) confirm that “access to financial markets facilitated by fintech innovations to trading platforms, along with ample free time, are significant determinants of retail-investor stock-market participation.” The importance of this development for market mechanics is underscored by van der Beck and Jaunin’s (2021) finding that “despite their negligible market share of 0.2% [...] Robinhood traders account for 10% of the cross-sectional variation in stock returns during the second quarter of 2020.”

This and other results underscore that the recent surge in retail trading, combined with the enormous growth of WallStreetBets, likely also affects the relationship between social media online activity and stock and option trading. Our goal is to provide updated evidence on that relationship and add a timely new angle to the existing literature.

## Data and methodology

### Data sources

We collect all WallStreetBets posts from the start of 2020 to the end of February 2021, in total more than 40 million, thereof 33.2 million in our trading hours-sample. For posts starting in January 2021 we got the posts directly from Reddit’s streaming API and for posts in 2020 we used the unofficial Pushshift API which ingests all Reddit posts (see Baumgartner et al. 2020, for more information on the Pushshift dataset and API). Comments are then sorted into GameStop-related and non-GameStop-related comments by the following procedure:

1. A comment is GameStop-related if “GameStop” or its ticker “GME” are mentioned in the comment.
2. If neither GameStop nor another stock symbol is mentioned in a comment, we search iteratively in the parent comment or post for the mention of a stock symbol or company name and classify the post as GameStop-related if the first parent stock symbol or company name mention is related to GameStop.

In total, we find that 4 million or about 12% of the comments in our sample period are GameStop-related with the share increasing from below 1% for most of the first half of 2020 to 60% at the peak end of January 2021.

We obtain consolidated stock TAQ trade data for GameStop from Interactive Brokers and consolidated option TAQ data from IVolatility. In our sample, the stock price of GameStop oscillated from a low of $2.57 on 2020/4/3 to a high of $508.02 on 2021/1/28. There were on average 9.8 million GameStop shares and 115.500 GameStop options (corresponding to 11.55 million shares) traded per day.

**Fig. 1:**
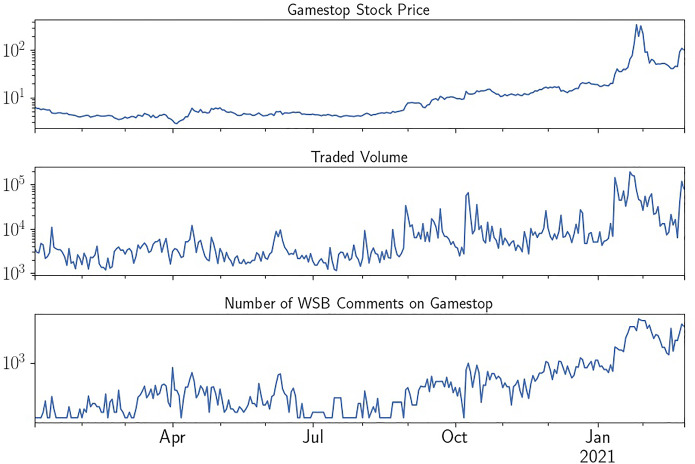
Logarithmic Chart of GameStop’s Stock Price, Traded Volume and Reddit WSB Comments. The Figure summarizes the development of GameStop’ stock price, the trading volume and the comments on WallStreetBets from January 2020 to March 2021

Figure 1 gives an overview of the development of GameStop’s share price, trading volume and GameStop-related WallStreetBets comments. Due to the enormous surge in the share price, as well as volume and comments, we use a logarithmic scale on the y-axis for all three measures. This strong trend introduces challenges for the empirical analysis, as it may cause spurious results with the strong growth clouding more nuanced relationships between variables. Thus, we employ multiple differencing and deseasonalizing approaches explained in Sect. 4.3 to check robustness of our results.

**Table 1:** Summary Statistics Raw Data

	N	Mean	Std	Q1	Median	Q3	Sum
*TRD*	3768	8360	28933	730	1280	2745	31,500,031
*Volume*	3768	849,218	2,150,092	142,794	269,348	581,467	3,199,852,975
*TO*	3768	49,017,415	230,993,725	649,855	1,608,373	6,449,112	184,697,618,639
*OTRD*	3767	1920	5909	70	198	818	7,231,546
*OVolume*	3767	9172	23,158	664	1915	5626	3,454,9251
*OTO*	3767	10,638,251	62,808,180	23929	104559	593,190	40,074,290,894
$RC_{ALL}$	20,014	1659	3473	577	1050	1783	33,202,416
$RC_{GME}$	20,014	204	1285	0	0	7	4,075,848
$RC_{ALL,TH}$	3681	3260	6441	1663	2089	2688	12,001,389
$RC_{GME, TH}$	3681	508	2527	0	1	13	1,870,951

Table 1 reports summary statistics for our raw data. Consolidated Stock Tick data is obtained from TAQ and consolidated Option Tick data from Ivolatility. *TRD* denotes trade count, *Volume* trade size and *TO* turnover (size*price). A preceding *O* denotes option trade data. Reddit comment statistics are given for all stocks (*ALL*) and GameStop-specific (*GME*), *TH* denotes comments during our trading hours sample. Data is then sorted into 30-min bins corresponding to 13.30-min bins for each full trading day in the sample. The sample period is January 2020–March 2021

Summary statistics on our raw data can be found in Table 1. We divide our sample period into 3768 30-min windows with each full trading day containing 13 of these windows (we follow e.g., Sun et al. 2016; Gao et al. 2018; Farrell et al. 2020, with the use of 30-min intraday windows). The first three rows show statistics related to the trading volume. *TRD* denotes the number of trades in each 30-min window; the dataset includes 31.5 million trades in total with an average of 8,360 during each 30-min period. As almost all our variables, this is heavily skewed due to the logarithmic rise of GameStop’s price and trading volume in early 2021, evidenced by the large difference between mean and median (1,280 trades). The next variable, *Volume* describes the number of shares traded, with a mean of 819,218 shares (which equals about 100 shares per trade on average) and a total of 3.2 billion shares traded. The turnover, *TO* is calculated as trade size*price and more important for our analysis than in comparable settings due to the huge price changes. Whereas turnover and volume are almost exchangeable for empirical analysis in most settings, the huge short-term price swings of the GameStop shares cause these variables to drift apart considerably. The average turnover is 49.0 million USD which equals a total turnover of 184.7 billion USD.

The following three rows with a preceding *O* show the same measures for GameStop-related option trading. We have a total of 7.2 million option trades with 34.5 million traded options in total and a turnover of 40.1 billion USD in our sample. The average option turnover in each 30-min window is 10.1 million USD, about one-fifth of the stock turnover. As a myriad of different options are traded and the option price depends on several parameters, there is no direct connection between volume and (total) turnover for options in the aggregated dataset.

In the last four rows, summary statistics for WallStreetBets are shown. $RC_{ALL}$ contains measure for all comments while GameStop-specific comments are denoted as ($RC_{GME}$). The last two rows (*TH*) limit comments to our trading hours sample, with a total of 12.0 million comments on our sample, thereof 1.9 million related to GameStop. On average there are 3,260 comments in each 30-minute window with 508 or almost 16% on GameStop; however this is also heavily skewed towards the end of our sample period.

### Variables used in the empirical analysis

Table 2 gives summary statistics on the variables used in our empirical analysis. As we do not conduct a broad cross section analysis but a case study focused on a specific stock with a highly unusual and very dynamic trading pattern and price and volume development, a single measure is not sufficient to shed light on the connection between Reddit posts and trading activity. Thus, we include multiple measures for (retail) trading activity and volume in our analysis and use a variety of control variables.

**Table 2:** Summary statistics regression variables

	N	Mean	Std	Q1	Median	Q3
Reddit comments & Trading volume variables (log)						
*lRC*	3681	1.7219	2.4276	0.0000	0.6931	2.6391
*lTO*	3768	14.7884	1.9444	13.3845	14.2907	15.6795
*lvolume*	3768	12.6886	1.1832	11.8692	12.5038	13.2733
*lOTO*	3767	11.9381	2.6064	10.0829	11.5575	13.2933
*lTRD*	3768	7.4850	1.3421	6.5944	7.1558	7.9179
Retail trading volume variables (log)						
*lRTO*(*OL*)	3768	13.5384	2.1340	12.0099	13.0048	14.4825
*lRTO*(*ST*)	3768	14.0603	1.5604	12.9279	13.6979	14.7784
*lROTO*(*OC*)	3767	9.2263	2.9178	7.0605	8.5618	10.7882
*lRTO*(*MR*)	3768	12.9670	2.1806	11.4287	12.4358	14.0506
Retail trading proportion variables						
*RTP*(*OL*)	3768	0.3046	0.1082	0.2342	0.2897	0.3510
*RTP*(*ST*)	3768	0.5253	0.1887	0.3931	0.5305	0.6654
*RTP*(*OC*)	3767	0.0933	0.0745	0.0430	0.0771	0.1241
*RTP*(*MR*)	3768	0.3868	0.1345	0.3056	0.3910	0.4709
Return and volatility variables						
*R*	3764	0.0007	0.0409	$-$0.0089	0.0000	0.0085
|*R*|	3764	0.0171	0.0371	0.0036	0.0087	0.0180
$AR(\beta _{ges})$	3764	0.0006	0.0405	$-$0.0084	$-$0.0004	0.0077
$AR(\beta _{30})$	3739	0.0006	0.0398	$-$0.0087	$-$0.0001	0.0078
$AR(\beta _{7})$	3739	0.0006	0.0384	$-$0.0088	-0.0002	0.0076
*mroibvol*	3768	$-$0.0109	0.3128	$-$0.1912	$-$0.0084	0.1594
*IVOL*	3691	0.0225	0.0315	0.0099	0.0135	0.0215

This table reports summary statistics for the variables used in the empirical analysis. *lRC* denotes the log number of Reddit Comments on GameStop during a 30-minute window. *lTO* is the log share turnover; *lVolume*, the logarithm of number of shares traded; *lOTO* the log option turnover and *lTRD*, the log number of share trades. In the next section, *lRTO*(*OL*) is the log retail share turnover measured by oddlot trades; *lRTO*(*ST*) is the log retail share turnover measured by small trades below USD 5,000; *lROTO*(*OC*) is the log retail option turnover, measured by one-contract option trades and *lRTO*(*MR*) the log retail share turnover, measured by marketable retail orders. The *RTP* measures in the third section denote the respective Retail Trading Proportion (retail turnover divided through total turnover). The last section contains return and volatility-related variables. *R* is the log return during one 30-min window, |*R*| the absolute return, *AR* denotes the abnormal return (market model) with $\beta$ calculated either for the whole sample or with a rolling 30- or 7-day window. *mroibvol* is the marketable retail order imbalance introduced by Boehmer et al. (2021) and *IVOL* the idiosyncratic volatility (market model, 7-day rolling volatility of residuals).The sample period is January 2020–March 2021

The first five columns include the Reddit comments on GameStop (*lRC*) and four different measures for general stock and option volume, as introduced in Sect. 4.1. In the second segment of Table 2, variables relating specifically to the retail stock and option turnover are given. As these measures contain only the turnover of trades classified as retail trades by different proxies, they are always subsets of the total stock or option turnover and thus smaller than *lTO*, respectively, *lOTO*. The retail classifications are defined as follows:

1. *lRTO*(*OL*) classifies all odd-lot trades as retail trades:
  $$\begin{aligned} lRTO(OL)=log(Turnover\; where\; Volume\;mod\;100 != 0) \end{aligned}$$
  This identification is one of the oldest and most established ones and follows e.g., Dyl and Maberly (1992). However, more recently O’Hara et al. (2014) and others warned that odd-lot trading, while still often used by retail traders, is increasingly caused by high frequency or algorithmic traders. As these kinds of traders are less likely in very-high volatility environments like GameStop in our sample period and odd-lot trading is still widely used as a proxy for retail trading, we incorporate odd-lots in our analysis.
2. The second measure, *lRTO*(*ST*), refers to small trades as main criterion. Here we follow e.g., Barber et al. (2006) and Han and Kumar (2013), who use a trade size of $ 5,000 as cut-off value for a classification as retail trade.
  $$\begin{aligned} lRTO(ST)=log(Turnover\; where\; Turnover<5000\,\, USD) \end{aligned}$$
  Han and Kumar (2013) confirm that their definition “closely captures the preferences and trading activities of retail investors” by comparing it with “actual retail holdings and trading data from a broker.” However, similar limitations as for *lRTO*(*OL*) apply.
3. Our third measure *lROTO*(*OC*) is option-based and thus avoids many of the shortfalls of *lRTO*(*OL*) and *lRTO*(*OL*) as automated trading is less prevalent on the option market, mainly due to lower liquidity and bigger spreads. It is based on the observation that retail traders mostly trade single option contracts (one contract corresponds to 100 shares) while institutional traders who use options e.g., to hedge positions rarely trade single contracts:
  $$\begin{aligned} lROTO(OC)=log(Option\;Turnover\; where\; Volume=1) \end{aligned}$$
  Retail option trading is a novel phenomenon and we are—to our knowledge—the first to introduce this option-based measure. While Battalio et al. (2004) already wrote that they “examine one-contract trades separately to isolate retail orders more confidently,” a more recent cross-sectional analysis is still missing in the scientific literature. However, research by brokers, e.g., Goldman Sachs, shows that retail option trading and especially one-contract trading increased sharply since the beginning of 2020 with one-contract trades now accounting for 13% of total option volume and even more for popular stocks.^10^ Our results indicate that there is indeed a significant relationship between Reddit posts and one-contract option turnover; however broader cross-sectional research is needed to confirm our GameStop-focused results and confirm whether this measure can be generalized to identify retail trading.
4. Finally, *lRTO*(*MR*) identifies marketable retail orders as laid out by Boehmer et al. (2021):
  $$\begin{aligned} lRTO(MR)= & {} log(Turnover\; where\; (Price\mod \;0.01)\;\\&*100\; in\; ]0,0.4]\; or\; [0.6,1[\;) \end{aligned}$$
  Trades are classified as retail trades if the TAQ data indicates that they have been reported through a FINRA-facility and are priced just below a round penny (fraction of a cent between 0.6 and 1) or just above a round penny (fraction of a cent between 0 and 0.4). While this classification captures retail trades reliably due to the regulatory rules around sub-penny price improvements and the increasing internalization of orders by retail brokers, it omits limit trades which are not marketable and all trades that are routed to exchanges.

The *RTP* measures in the third section then denote the respective Retail Trading Proportion (retail turnover divided through total turnover). While the non-option-based measures indicate retail trading proportions of 30–52%, the option-based measure only shows a mean retail trading proportion of 9%. While these measures are calculated as a fraction, their development over time shows a clear trend and indicates non-stationarity.

**Fig. 2:**
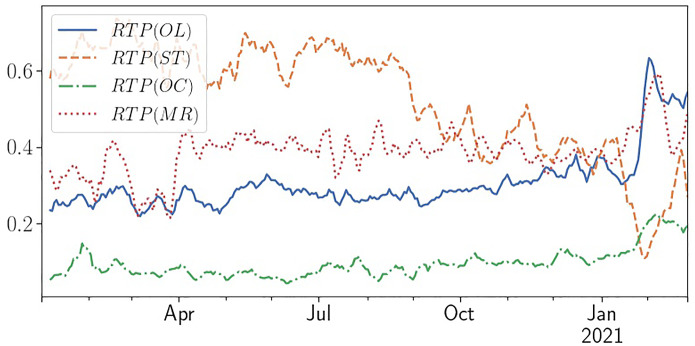
Daily Development of *RTP*, measured as Proportion of Trade Volume (Rolling Average over 5 Days). The Figure summarizes the development of the Retail Trading Proportion in GameStop shares and options according to different proxies introduced in Sect. 4.2 from January 2020 to March 2021

In Fig. 2, one can see that most measures slowly increase in the second half of 2020 and peak end of January 2021, with the increases most pronounced for *RTP*(*OL*), while *RPT*(*MR*) changes least. The decrease for *RTP*(*ST*) is expected, as this measure is directly and inversely related to the price of the shares.

The last section contains return and volatility-related variables that are used for the analysis of informativeness and as control variables. *R* is the log return of GameStop shares during one 30-minute window and |*R*| the log absolute return. The *AR* variables denote the abnormal return of GameStop shares and are calculated using the simple market model:

$$\begin{aligned} AR_{GME,t}=R_{GME,t}-(\beta _{GME,t}R_{m,t}-R_{rf,t}) \end{aligned}$$

For the first variant ($AR(\beta _{ges})$), beta is calculated for our whole sample and equates to approximately 1.1. As this estimate seems to be very low given the huge price fluctuations, we also calculate $AR(\beta _{30})$ and $AR(\beta _{7})$ with the rolling beta over the previous 30, respectively, 7 days (in calendar time). While the 7-day beta for example swings between a minimum of -20 and a maximum of +13, its mean of 1.3, 25% quantile of 0.7 and 75% quantile of 2.5 are in the more usual territory and the summary statistics for abnormal returns indicate only negligible changes. The next variable, *mroibvol*, is the marketable retail order imbalance introduced by Boehmer et al. (2021), defined as the difference of turnover of marketable retail buy orders and retail sell orders divided through the total turnover of marketable retail orders. *IVOL*, used as additional control variable, is the idiosyncratic volatility (calculated with the market model on a 7-day rolling window).

### Differencing and deseasonalizing approaches

**Fig. 3:**
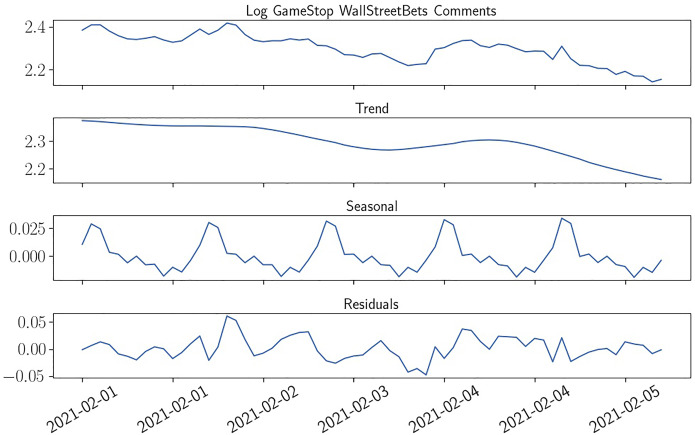
STL Decomposition of Log Reddit Comments. The Figure shows the effect of LOESS-based STL decomposition of a timeseries in trend, seasonal and residual components on the example of log WallStreetBets comments in the week from 2021/02/01 to 2021/02/05

Due to the aforementioned challenges with non-stationarity and logarithmic increase in comment and trading measures, we apply three different differencing and deseasonalizing approaches to our Reddit comment and trading activity data:

1. First, in our main specification we difference out the variable’s levels with its level from one week prior. That means, when e.g., predicting the log trading volume we don’t e.g., predict the level on Monday, the 25th of January 2021 between 9:30 AM and 10:00 AM but the difference between the log trading volume in that period and the log trading volume one week prior (in this case Monday, 18th of January 2021 between 9:30 AM and 10:00 AM). The same transformation is applied to the Reddit comments and non-stationary control variables (for the preceding 30-min window). This approach succeeds in achieving stationarity (as evidenced by non-significant Intercepts) and at the same time alleviates concerns regarding time trends and seasonality due to daily patterns during our 30-minute windows. This transformation is denoted with *WCHG* (weekly change) in our results. However, due to the lag of one week, one could argue that short-term trends could still distort results.
2. Thus, we also show results that are differenced out with the preceding 30-minute window, denoted with *CHG* (change). We suppose that this approach includes a lot of noise due to seasonality of intraday and intraweek returns and Reddit comments but feel that the first difference is the most natural way of measuring changes and thus should be included in the analysis.
3. In addition to both differencing approaches described above, we explicitly try to remove seasonal and trend components in a third specification. For this, we use the seasonal-trend decomposition procedure based on LOESS (locally estimated scatterplot smoothing) introduced by Cleveland et al. (1990). This approach is well suited to split timeseries that contain nonlinearities and jumps into seasonal, trend and residual components. For our transformed timeseries that are denoted with *STL*, seasonal and trend components are subtracted so that only the residuals remain. While this transformation introduces look-ahead bias and should not be used to claim exploitable market anomalies, it serves us well to remove any short-term trend that could still affect results of the differencing approaches and check the robustness of our previous results. An example of the effect of STL decomposition on log Reddit comments is given in Fig. 3.

In addition to these transformations, we add half-hour and weekday controls as well as a continuous monthly trend control to our regression specifications (13$\times$5$\times$14).

## Results

In this section, we discuss the results of our regression analyses for the relationship between Reddit comments on GameStop and the firms general trading volume, the retail trading volume and proportion and excess returns.

### Regression analysis of the effect of comments on trading volume

For our main analysis, we explore the potential predictive effect of Reddit comments on GameStop trading volume using the following regression

1 $$\begin{aligned} lVolume_{30m,t}= & {} Intercept+\beta _{1}{lVolume_{30m,t-1}}+\beta _{2}{lVolume_{1d,t-1}}\nonumber \\&+\beta _{3}{lRC_{30m,t-1}}+\beta _{4}{lRC_{1d, t-1}}\nonumber \\&+\beta _{5}{|R|_{30m,t-1}}+\beta _{6}{|R|_{1d, t-1}}\nonumber \\&+\beta _{7}{R_{30m,t-1}}+\beta _{8}{R_{1d, t-1}}\nonumber \\&+\beta _{9}{IVOL_{30m,t-1}}+\beta _{10}{IVOL_{1d, t-1}}\nonumber \\&+{Time}+{Trend}+\epsilon \end{aligned}$$

where the dependent variable *lVolume* stands for our different variations of trade volume measures (turnover, volume, option turnover, trade count for total trade activity) and the change in the log number of Reddit comments *lRC* in the preceding 30-minute window as main variable of interest. The dependent variable, its lagged values and the variable of interest are transformed as described in Sect. 4.3. The absolute log return |*R*|, the log return *R* and the idiosyncratic volatility *IVOL* are used as additional control variables on the right-hand side. For all right-hand-side variables, we add controls for the preceding trading day (in addition to the preceding 30-minute window) as well, denoted with 1*d*^11^. As we are less interested in the results for control variables, we accept some risk of multicollinearity between controls in exchange for higher confidence in the results for our comment-related dependent variables, achieving a higher overall R-squared. For all independent variables, we subtract the mean and divide through the standard deviation to standardize them and make coefficients comparable. In addition, we add half-hour and weekday as well as a continuous monthly trend variable to control for time and trend fixed effects (13$\times$5$\times$14).

Standard errors are corrected for potential heteroskedasticity and autocorrelation, using the Newey and West (1987) methodology with 13 lags which equals one normal trading day of 30-minute periods and significance is denoted by * for *p* < 0.1, ** for *p* < 0.05 and *** for *p* < 0.01.

**Table 3:** Effect of Reddit comments on GameStop trading volume—weekly differences

Trading volume, weekly differences								
	$lTO_{wchg}$		$lVolume_{wchg}$		$lOTO_{wchg}$		$lTRD_{wchg}$	
	Coef.	t-stat	Coef.	t-stat	Coef.	t-stat	Coef.	t-stat
$lRC_{wchg,30m}$	**0**.1595***	**8.89**	**0**.1558***	**9.17**	**0**.2690***	**8.88**	**0**.0778***	**6.76**
$lRC_{wchg,1d}$	$-$**0**.**0088**	$-$**0**.**38**	$-$**0**.**0091**	$-$**0.43**	**0**.**0008**	**0.02**	**0**.**0090**	**0.59**
$DepVar_{lagged,30m}$	0.6078***	22.31	0.5824***	22.56	0.4119***	17.78	0.7098***	31.70
$DepVar_{lagged, 1d}$	0.1037***	3.21	0.1115***	3.60	0.1889***	5.35	0.0594**	2.29
$|R|_{30m}$	0.0202	0.95	0.0215	1.01	0.0626	1.52	0.0167	1.09
$|R|_{1d}$	$-$0.0300	$-$0.35	$-$0.1106	$-$1.35	$-$0.3027**	$-$1.96	$-$0.0200	$-$0.37
$R_{30m}$	0.0514***	3.86	0.0341***	2.80	0.0592***	3.57	0.0354***	3.46
$R_{1d}$	0.0725***	3.39	0.0219	1.23	0.0876***	2.83	0.0426***	3.54
$IVOL_{30m}$	0.0588	1.42	0.0531	1.35	0.1990***	3.15	0.0327	1.56
$IVOL_{1d}$	$-$0.0325	$-$0.45	0.0156	0.23	0.1268	0.90	$-$0.0120	$-$0.26
*Intercept*	0.0012	0.02	$-$0.0016	$-$0.02	0.0068	0.04	0.0316	0.57
Time FE & Trend	*yes*		*yes*		*yes*		*yes*	
Adj. $R^{2}$	0.62		0.52		0.39		0.70	
Obs	3310		3310		3306		3310	

*$$p<0.1$$, **$$p<0.05$$, ***$$p<0.01$$

This table reports the regression estimates for the effect of Reddit comments on trading measures in 30-minute windows. The dependent variables are: (i) *lTO*, the log share turnover; (ii) *lVolume*, the logarithm of number of shares traded; (iii) *lOTO*, the log option turnover and (iv) *lTRD*, the log number of share trades. The independent variables of interest are: (i) $lRC_{30m}$ which denotes the log number of Reddit Comments on GameStop during the preceding 30-min window and (ii) $lRC_{1d}$, which is defined in the same way as (i) but counts all comments made on the previous day instead of the preceding 30-min window. For both, the dependent variables and the independent variables of interest, we employ weekly differences (*wchg*) to achieve stationarity and avoid trend and seasonality bias. Additionally, the following control variables are used: (i) *DepVar*, a lagged measure of the dependent variable for the preceding 30-minute window and the previous day; (ii) |*R*|, the absolute return of GameStop shares; (iii) *R*, the return of GameStop shares and (iv) *IVOL*, the idiosyncratic volatility. All independent variables are measured at least one period ahead of the dependent variable and are standardized with zero mean and a standard deviation of one to make coefficients comparable. Additionally, we add Time Fixed Effects (for all 30-minute windows and weekdays) & a trend variable (counting months from start of sample). We use the Newey and West (1987) methodology with 13 lags to correct standard errors for potential heteroskedasticity and autocorrelation. The sample period is January 2020–March 2021; independent variables of interest are shown in bold

Results for our main specification, showing the relationship between Reddit comments and different trading volume measures applying the weekly differences approach, can be found in Table 3. Coefficients for all volume measures are significantly positive (*p* < 0.0001) with t-values ranging from 6.76 for the number of trades to 9.17 for the trade volume. Coefficients can be interpreted as follows: the standardized coefficient of $lRC_{wchg,30m}$ for the log turnover *lTO* of approximately 0.16 means, that for a one standard deviation change of the log number of Reddit comments on GameStop in a 30-minute window compared to the previous week, the difference in log turnover vs the previous week in the following 30-minute window increases by 0.16. A standard deviation of 1.32 for $lRC_{wchg,30m}$ equals an increase of $exp(1.32)-1=272\%$ in the weekly difference of comments that would be followed by a $exp(0.16)-1=17\%$ weekly increase in turnover in the following 30-minute window. Surprisingly, the effect is largest for option turnover with an increase of 30% for one standard deviation in comment change while the coefficient for the number of trades is considerably smaller, leading to the conclusion that the trade number is less influenced by Reddit comments than total turnover. In contrast to the difference of comments in the preceding 30-min window, the difference of comments on the previous trading day versus one week ago is not significant for the change in trading volume and has even negative coefficients for some variants.

For the control variables, we find that the lagged versions of the respective volume measure have significant positive coefficients in all cases, hinting at autocorrelation for the weekly difference in trading volume. While coefficients and t-values for the previous 30-min window are very high, the trading volume difference on the previous day in the fourth row is less relevant with smaller coefficients and t-values for future trading volume change than the change in Reddit comments in the preceding 30-min window. The only other control variables that are consistently significant predicting the change in trading volume are lagged measures of GameStop’s log return (with the exchange of previous-day return for *lVolume*). Interestingly, the 30-min-lagged idiosyncratic volatility, which is calculated with market-model abnormal returns for stock prices, does have a significant predictive effect for the difference in GameStop option turnover but not for any of the stock-based turnover measures which could be explained by additional information from stock market activity that is already absorbed for stock-related measures.

R-squared is unsurprisingly lowest for the change in option turnover with 0.39 and reaches 0.70 for the change in the number of trades, demonstrating a high explanatory power of the model.

Results for the 30-min changes in log trading volume in Table 4 mostly confirm the impression of the first results.

**Table 4:** Effect of Reddit comments on GameStop trading volume - half-hour differences

	$lTO_{chg}$		$lVolume_{chg}$		$lOTO_{chg}$		$lTRD_{chg}$	
	Coef.	t-stat	Coef.	t-stat	Coef.	t-stat	Coef.	t-stat
$lRC_{chg,30m}$	**0**.0229**	**2.37**	**0**.0228**	**2.37**	**0**.0315*	**1.86**	**0**.0187***	**2.76**
$lRC_{chg,1d}$	**0**.**0003**	**0.05**	**0**.**0010**	**0.15**	$-$**0.0015**	$-$**0.13**	−**0.0005**	−**0.11**
$DepVar_{lagged,30m}$	$-$0.3651***	$-$21.37	$-$0.3635***	$-$21.21	$-$0.4036***	$-$20.88	$-$0.3071***	$-$16.55
$DepVar_{lagged,1d}$	$-$0.0289***	$-$2.81	$-$0.0324***	$-$3.14	$-$0.0201*	$-$1.67	$-$0.0282***	$-$2.85
$|R|_{30m}$	0.0359***	3.46	0.0308***	2.92	0.0225	1.54	0.0280***	3.18
$|R|_{1d}$	0.0356	1.04	0.0359	1.12	$-$0.0122	$-$0.25	0.0231	0.80
$R_{30m}$	0.0396***	2.66	0.0192	1.35	0.0549***	2.70	0.0229*	1.80
$R_{1d}$	0.0013	0.17	$-$0.0007	$-$0.10	$-$0.0026	$-$0.27	0.0025	0.43
$IVOL_{30m}$	$-$0.0583***	$-$4.10	$-$0.0576***	$-$4.50	$-$0.0285	$-$1.60	$-$0.0456***	$-$3.70
$IVOL_{1d}$	$-$0.0171	$-$0.60	$-$0.0159	$-$0.59	0.0073	0.17	$-$0.0073	$-$0.29
*Intercept*	0.3421***	6.76	0.3349***	6.67	0.4916***	6.44	0.0909**	2.13
Time FE & Trend	*yes*		*yes*		*yes*		*yes*	
Adj. $R^{2}$	0.29		0.30		0.19		0.37	
Obs	3554		3554		3551		3554	

*$$p<0.1$$, **$$p<0.05$$, ***$$p<0.01$$

This table reports the regression estimates for the effect of Reddit comments on trading measures in 30-minute windows. The dependent variables are: (i) *lTO*, the log share turnover; (ii) *lVolume*, the logarithm of number of shares traded; (iii) *lOTO*, the log option turnover and (iv) *lTRD*, the log number of share trades. The independent variables of interest are: (i) $lRC_{30m}$ which denotes the log number of Reddit Comments on GameStop during the preceding 30-minute window and (ii) $lRC_{1d}$, which is defined in the same way as (i) but counts all comments made on the previous day instead of the preceding 30-min window. For both, the dependent variables and the independent variables of interest, we apply first-differences (*chg*) to achieve stationarity. Additionally, the following control variables are used: (i) *DepVar*, a lagged measure of the dependent variable for the preceding 30-minute window and the previous day; (ii) |*R*|, the absolute return of GameStop shares; (iii) *R*, the return of GameStop shares and iv) *IVOL*, the idiosyncratic volatility. All independent variables are measured at least one period ahead of the dependent variable and are standardized with zero mean and a standard deviation of one to make coefficients comparable. Additionally, we add Time Fixed Effects (for all 30-min windows and weekdays) & a trend variable (counting months from start of sample). We use the Newey and West (1987) methodology with 13 lags to correct standard errors for potential heteroskedasticity and autocorrelation. The sample period is January 2020–March 2021; independent variables of interest are shown in bold

Differencing volume-related and comment measures against the previous 30-minute window can’t get rid of seasonality and leaves a significant intercept in our model. Coefficients for all trading volume measures are still significantly positive, albeit with considerably smaller coefficients and t-values. In this case, the coefficient of 0.0187 for the effect of a change of one standard deviation in the log number of comments in the previous 30-minute window for the number of trades means that an increase of approximately 90% in Reddit comments compared to the previous 30 minutes predicts a 2% increase in the number of trades in the following 30-min window. While overall small, the effect size and direction confirm prior results from us and others. In contrast, the one-day change of comments on the previous day does not have any significant impact on the following change in trade volume.

Notably, the lagged dependent variable is highly significant for all trading volume measures but shows a negative sign in contrast to the weekly differences. That result has several important implications: First, this could be interpreted as some kind of reversion to the mean (e.g., return to normal trading volume after periods of very high trading volume). Second, this result could also indicate a complex and nonlinear trend profile over different time horizons. While a high change of trading volume versus the volume one week ago predicts a positive weekly change in the following period as well, a positive change of trading volume versus the volume 30 minutes ago predicts a negative 30-min change in the following period. Third, while the sign changes for the impact of lagged trading volume, the sign for the impact of Reddit comments stays positive. This could mean that the attention mechanism is more stable, with an increase in the number of comments preceding an increase in trading volume regardless the time horizon. This also somewhat alleviates concerns of reverse causality (e.g., trading volume spikes that drive comment numbers which in turn increase trading volume again at a later time), as in this case we would expect the signs of both variables to behave similarly. However, despite this interesting result, we cannot argue that this underscores a causal relationship as there are multiple alternative explanations for this behavior.

Change in Idiosyncratic volatility over the 30-min window has a significant negative relationship to trading volume change in the following window for all measures except option turnover, probably also indicating some kind of “cooling-down” effect after short-term volatility spikes. Unsurprisingly, the R-squared is considerably smaller for all variables in Table 4, ranging between 0.19 for option turnover and 0.37 for number of trades.

As a robustness check, Table 5 shows the results for STL decomposition of comments and trading volume as described in Sect. 4.3. Stripped of their trend and seasonal components, we analyze whether the remaining residuals in comment count can explain the residuals of trade volume in the following 30-min window. The results have serious caveats due to lookahead bias, possible interaction effects and loss of valuable information due to the decomposition procedure and should not be interpreted in isolation. However, using them as an additional robustness check to validate previous results and as another, non-conclusive piece of potential evidence for a causal effect of Reddit comments on trading activity seems beneficial, as this setup doesn’t require to take a difference that could introduce additional noise.

**Table 5:** Effect of Reddit comments on GameStop trading volume - seasonal-trend decomposition

Trading volume, seasonal-trend decomposition								
	$lTO_{STL}$		$lVolume_{STL}$		$lOTO_{STL}$		$lTRD_{STL}$	
	Coef.	t-stat	Coef.	t-stat	Coef.	t-stat	Coef.	t-stat
$lRC_{STL,30m}$	**0**.0012**	**2.56**	**0**.0013**	**2.44**	**0**.0021*	**1.69**	**0**.0011*	**1.90**
$lRC_{STL,1d}$	$-$0.0007*	$-$**1.89**	$-$0.0007*	$-$**1.78**	$-$**0.0009**	$-$**1.05**	$-$0.0008*	$-$**1.67**
$DepVar_{lagged,30m}$	0.2012***	7.24	0.1961***	7.41	0.1496***	6.66	0.3039***	10.15
$DepVar_{lagged,1d}$	$-$0.1650***	$-$2.97	$-$0.1731***	$-$2.99	$-$0.1562**	$-$2.40	$-$0.1042**	$-$2.38
$|R|_{30m}$	0.0026***	2.96	0.0033***	3.21	0.0029**	2.48	0.0037***	3.45
$|R|_{1d}$	$-$0.0037	$-$1.62	$-$0.0045*	$-$1.70	$-$0.0064	$-$1.40	$-$0.0064**	$-$2.14
$R_{30m}$	0.0012*	1.84	0.0006	0.85	0.0019**	2.48	0.0012	1.47
$R_{1d}$	$-$0.0008*	$-$1.78	$-$0.0012**	$-$2.47	$-$0.0005	$-$0.71	$-$0.0012**	$-$2.19
$IVOL_{30m}$	0.0004	0.46	0.0006	0.59	0.0017	1.23	0.0006	0.52
$IVOL_{1d}$	0.0023	1.40	0.0025	1.26	0.0045	1.13	0.0043*	1.91
*Intercept*	0.0009	0.41	0.0004	0.16	0.0096	1.37	0.0028	0.85
Time FE & Trend	*yes*		*yes*		*yes*		*yes*	
Adj. $R^{2}$	0.07		0.07		0.04		0.13	
Obs	3600		3600		3598		3600	

*$$p<0.1$$, **$$p<0.05$$, ***$$p<0.01$$

This table reports the regression estimates for the effect of Reddit comments on trading measures in 30-minute windows. The dependent variables are: (i) *lTO*, the log share turnover; (ii) *lVolume*, the logarithm of number of shares traded; (iii) *lOTO*, the log option turnover and (iv) *lTRD*, the log number of share trades. The independent variables of interest are: (i) $lRC_{30m}$ which denotes the log number of Reddit Comments on GameStop during the preceding 30-min window and (ii) $lRC_{1d}$, which is defined in the same way as (i) but counts all comments made on the previous day instead of the preceding 30-minute window. For both, the dependent variables and the independent variables of interest, we apply seasonal-trend decomposition based on LOESS (*STL*) to remove trend and seasonality components. Additionally, the following control variables are used: (i) *DepVar*, a lagged measure of the dependent variable for the preceding 30-min window and the previous day; (ii) |*R*|, the absolute return of GameStop shares; (iii) *R*, the return of GameStop shares and iv) *IVOL*, the idiosyncratic volatility. All independent variables are measured at least one period ahead of the dependent variable and are standardized with zero mean and a standard deviation of one to make coefficients comparable. Additionally, we add Time Fixed Effects (for all 30-min windows and weekdays) & a trend variable (counting months from start of sample). We use the Newey and West (1987) methodology with 13 lags to correct standard errors for potential heteroskedasticity and autocorrelation. The sample period is January 2020–March 2021; independent variables of interest are shown in bold

While the coefficients are very small and the t-values between 1.69 and 2.56 are only barely significant for all trade volume measures, the small but consistently positive predictive effect of residual Reddit comments on future residual trade volume confirm the picture of our previous results. Lagged values of trade volume are also significant with considerably higher coefficients in this model, which is somewhat expected when using level values with imperfect decomposition. However, while for Table 3 with weekly differences all lagged versions of the dependent variable were positive and for Table 4 all were negative, in this case the residual trade volume in the preceding 30 minutes has a positive effect on following trade volume while the residual trade volume on the previous day has a negative effect. This further underlines the complex time-dependent autocorrelated profile of the trade volume and strengthens our suspicion of nonlinear behavior.

### Regression analysis of the effect of comments on retail trading volume and proportion

Next, we turn to the trading volume specifically caused by Retail investors. To differentiate between retail and non-retail trades, we use four different proxies described in Sect. 4.2. In addition to the control variables used for previous results in Sect. 5.2, we add total (stock) turnover *lTO* as additional control variable. The rest of the model remains unchanged.

**Table 6:** Effect of Reddit Comments on GameStop retail trading volume—weekly differences

Retail Trading Volume, Weekly Differences								
	$lRTO(OL)_{wchg}$		$lRTO(ST)_{wchg}$		$lROTO(OC)_{wchg}$		$lRTO(MR)_{wchg}$	
	Coef.	t-stat	Coef.	t-stat	Coef.	t-stat	Coef.	t-stat
$lRC_{wchg,30m}$	**0**.1471***	**7.65**	**0**.1061***	**7.59**	**0**.1281***	**5.83**	**0**.1578***	**7.23**
$lRC_{wchg,1d}$	**0**.**0248**	**0.97**	$-$**0.0111**	$-$**0.62**	**0**.**0389**	**1.42**	**0**.**0357**	**1.44**
$DepVar_{lagged,30m}$	0.2245***	5.73	0.6902***	18.45	0.4843***	15.74	0.4255***	14.96
$DepVar_{lagged,1d}$	0.0717	0.77	0.1042	1.26	0.1078**	2.46	0.0858	1.55
$lTO_{wchg,30m}$	0.4294***	8.86	$-$0.0270	$-$0.81	0.2442***	6.54	0.2836***	7.02
$lTO_{wchg,1d}$	0.0142	0.17	$-$0.0022	$-$0.03	$-$0.0187	$-$0.35	0.0048	0.08
$|R|_{30m}$	0.0269	1.41	0.0137	0.82	0.0472**	2.14	0.0381	1.44
$|R|_{1d}$	$-$0.0392	$-$0.38	$-$0.0361	$-$0.55	$-$0.1651	$-$1.29	$-$0.1784*	$-$1.86
$R_{30m}$	0.0502***	3.45	0.0425***	3.69	0.0445***	3.22	0.0359**	2.40
$R_{1d}$	0.0782***	3.19	0.0445***	2.85	0.0515**	2.05	0.0300	1.35
$IVOL_{30m}$	0.0925**	2.10	0.0268	0.90	0.0891**	2.42	0.0827**	2.01
$IVOL_{1d}$	$-$0.0044	$-$0.05	0.0018	0.03	0.1028	0.92	0.0906	1.14
*Intercept*	0.0373	0.45	0.0126	0.19	0.1279	0.94	0.0404	0.44
Time FE & Trend	*yes*		*yes*		*yes*		*yes*	
Adj. $R^{2}$	0.59		0.65		0.57		0.59	
Obs	3310		3310		3306		3310	

*$$p<0.1$$, **$$p<0.05$$, ***$$p<0.01$$

This table reports the regression estimates for the effect of Reddit comments on retail trading measures in 30-min windows. The dependent variables are: i) *lRTO*(*OL*), the log retail share turnover measured by oddlot trades; ii) *lRTO*(*ST*), the log retail share turnover measured by small trades below USD 5,000; (iii) *lROTO*(*OC*), the log retail option turnover measured by one-contract option trades and (iv) *lRTO*(*MR*), the log retail share turnover, measured by marketable retail orders. The independent variables of interest are: (i) $lRC_{30m}$ which denotes the log number of Reddit Comments on GameStop during the preceding 30-min window and (ii) $lRC_{1d}$, which is defined in the same way as (i) but counts all comments made on the previous day instead of the preceding 30-minute window. For both, the dependent variables and the independent variables of interest, we employ weekly differences (*wchg*) to achieve stationarity and avoid trend and seasonality bias. Additionally, the following control variables are used: (i) *DepVar*, a lagged measure of the dependent variable for the preceding 30-minute window and the previous day; (ii)*lTO* the log of share turnover; (iii) |*R*|, the absolute return of GameStop shares; (iv) *R*, the return of GameStop shares and (v) *IVOL*, the idiosyncratic volatility. All independent variables are measured at least one period ahead of the dependent variable and are standardized with zero mean and a standard deviation of one to make coefficients comparable. Additionally, we add Time Fixed Effects (for all 30-min windows and weekdays) & a trend variable (counting months from start of sample). We use the Newey and West (1987) methodology with 13 lags to correct standard errors for potential heteroskedasticity and autocorrelation. The sample period is January 2020–March 2021; independent variables of interest are shown in bold

Table 6 shows the relationship between weekly differences of Reddit comments and retail turnover in the following 30-min window. In general, the results are very similar to results for the effect of Reddit comments on total turnover. While the coefficient for total turnover in Table 3 was 0.1595 with a t-value of 8.89, coefficients are slightly lower for stock-based retail turnover range from 0.1061 for the turnover of small trades below 5,000 USD (*lRTO*(*ST*)) to 0.1578 for FINRA marketable retail orders (*lRTO*(*MR*)) with t-values between 7.23 and 7.65. For option-based turnover *lROTO*(*OC*) the coefficient halves from 0.2690 to 0.1281 and the t-value decreases from 8.88 to 5.83 when only looking at one-contract trades. However, the slight decrease in effect strength could also be due to the addition of turnover as additional control variable and despite this, the relationship remains consistently positive and significant. With regard to the other control variables, the general picture is similar. Coefficients for the 30-min lagged dependent variable are smaller than in the total turnover model, also likely caused by the addition of total turnover as additional control and resulting collinearity. One notable exception is the previous-day lagged dependent variable—while we had a t-value of 3.21 for its effect on the weekly difference of total turnover in Table 3 (and 5.35 for total option turnover), the maximum t-value for its effect on future retail turnover is failing to reach significance at 1.55 for the stock-based measures and barely reaches significance for retail option turnover at 2.46. The addition of total turnover does not seem to be a reason, as all one-day lagged variants thereof don’t reach t-values greater than 0.35. This result could be an indication that trends for retail turnover are shorter-lived, so that previous-day retail volume has a smaller impact compared to total volume.

R-squared is also similar for retail stock-based turnover and slightly higher for one-contract option turnover compared to total option turnover, increasing from 0.39 to 0.57 (which also could be caused by the addition of stock turnover as control variable).

Results for retail turnover calculated with 30-min differenced variables (Table 11) and STL-decomposed timeseries (Table 12) also mirror respective results for total turnover closely and can be found in the Supplementary Material.

As this approach fails to separate a specific effect of Reddit comments on GameStop retail turnover, we next turn to the proportion of retail turnover to total turnover (*RTP* for Retail Trading Proportion) as dependent variable. As seen in Sect. 4.2, the retail trading proportion is rising during our sample but as it is calculated as a fraction, it does not exhibit exponential growth. The rest of our model remains unchanged.

**Table 7:** Effect of Reddit comments on GameStop retail trading proportion—weekly Differences

Retail trading proportion, weekly differences								
	$RTP(OL)_{wchg}$		$RTP(ST)_{wchg}$		$RTP(OC)_{wchg}$		$RTP(MR)_{wchg}$	
	Coef.	t-stat	Coef.	t-stat	Coef.	t-stat	Coef.	t-stat
$lRC_{wchg,30m}$	$-$0.0057**	$-$**2.29**	$-$0.0173***	$-$**5.09**	$-$**0.0015**	$-$**0.85**	$-$ **0.0035**	$-$**0.93**
$lRC_{wchg,1d}$	**0**.0091***	**2.82**	$-$**0.0023**	$-$**0.52**	**0**.**0026**	**1.10**	**0**.0153***	**3.49**
$DepVar_{t-1}$	0.1594***	7.23	0.2095***	8.12	0.0896***	3.94	0.2119***	7.91
$DepVar_{Day-1}$	0.2670***	4.43	0.1672***	3.47	0.0670	1.14	0.2244***	4.42
$lTO_{wchg,30m}$	$-$0.0080***	$-$2.85	$-$0.0226***	$-$4.79	$-$0.0046**	$-$1.99	0.0042	1.00
$lTO_{wchg,1d}$	$-$0.0018	$-$0.45	0.0135**	2.34	0.0003	0.11	0.0058	1.08
$|R|_{30m}$	0.0025	1.09	0.0006	0.29	0.0062***	4.07	0.0047*	1.70
$|R|_{1d}$	$-$0.0035	$-$0.19	$-$0.0173	$-$1.07	0.0027	0.29	$-$0.0545***	$-$3.01
$R_{30m}$	$-$0.0001	$-$0.04	$-$0.0040**	$-$2.07	$-$0.0006	$-$0.57	$-$0.0049*	$-$1.91
$R_{1d}$	0.0056	1.28	$-$0.0082**	$-$2.28	0.0011	0.52	$-$0.0121***	$-$3.10
$IVOL_{30m}$	0.0115*	1.92	$-$0.0081	$-$1.48	$-$0.0012	$-$0.33	0.0217***	4.70
$IVOL_{1d}$	0.0114	0.64	0.0176	1.30	0.0027	0.32	0.0423***	2.69
*Intercept*	$-$0.0005	$-$0.04	$-$0.0007	$-$0.04	0.0198	1.55	0.0034	0.19
Time FE & Trend	*yes*		*yes*		*yes*		*yes*	
Adj. $R^{2}$	0.13		0.16		0.03		0.10	
Obs	3310		3310		3306		3310	

*$$p<0.1$$, **$$p<0.05$$, ***$$p<0.01$$

This table reports the regression estimates for the effect of Reddit comments on the retail trading proportion in 30-minute windows. The dependent variables are: (i) *RTP*(*OL*), the log retail share turnover measured by oddlot trades; (ii) *RTP*(*ST*), the log retail share turnover measured by small trades below USD 5,000; (iii) *RTP*(*OC*), the log retail option turnover measured by one-contract option trades and (iv) *RTP*(*MR*), the log retail share turnover, measured by marketable retail orders. The independent variables of interest are: (i) $lRC_{30m}$ which denotes the log number of Reddit Comments on GameStop during the preceding 30-min. window and (ii) $lRC_{1d}$, which is defined in the same way as (i) but counts all comments made on the previous day instead of the preceding 30-minute window. For both, the dependent variables and the independent variables of interest, we employ weekly differences (*wchg*) to achieve stationarity and avoid trend and seasonality bias. Additionally, the following control variables are used: (i) *DepVar*, a lagged measure of the dependent variable for the preceding 30-minute window and the previous day; (ii)*lTO* the log of share turnover; (iii) |*R*|, the absolute return of GameStop shares; (iv) *R*, the return of GameStop shares and (v) *IVOL*, the idiosyncratic volatility. All independent variables are measured at least one period ahead of the dependent variable and are standardized with zero mean and a standard deviation of one to make coefficients comparable. Additionally, we add Time Fixed Effects (for all 30-min windows and weekdays) & a trend variable (counting months from start of sample). We use the Newey and West (1987) methodology with 13 lags to correct standard errors for potential heteroskedasticity and autocorrelation. The sample period is January 2020—March 2021; independent variables of interest are shown in bold

Trying to differentiate between the effect specifically on retail turnover, the results for the effect of the 1 week difference of log Reddit comments on the weekly difference in the retail trading proportion in the following 30-min window can be found in Table 7. The effect of the weekly change in Reddit comments on the change of the retail trading proportion in the following 30-min window is negative among all our retail proxies with very small coefficients and only significant for the small-trade retail proxy. For the weekly change of Reddit comments one day prior, we find a significant but small coefficient for its impact on the retail trading proportions based on odd-lots and marketable retail orders. There could be several explanations for these results (e.g., institutional turnover increasing faster than retail turnover after an increase in Reddit comments due to better ability to screen social activity and react in real-time or proxies failing to pick up parts of the short-term retail-driven turnover).

However, as also evidenced by the low R-squared values between 0.03 and 0.16, our model fails to explain the change in retail trading proportion and we are not able to find evidence for a significantly different effect of Reddit comments on turnover caused by different kinds of investors.^12^

### Regression analysis of the effect of comments on abnormal returns

While an impact of online activity on future trade volume is a good indicator for the effect of investor attention, a second important question is whether there is an effect of Reddit comments on (short term) abnormal returns, which could be an indicator for the informativeness of Reddit comments. If we find a significant positive effect of the change in the number of Reddit comments on abnormal returns for GameStop in the following 30-minute window, it would be more improbable that Reddit comments only facilitate noise trading with no contribution to price finding. However, please note that our analysis is limited to one 30-minute window and a longer horizon would be needed for a conclusive analysis of informativeness.

**Table 8:** Effect of Reddit comments on GameStop abnormal return

Abnormal Return and Marketable Order Imbalances								
	$AR(\beta _{ges})$		$AR(\beta _{30})$		$AR(\beta _{7})$		$mroibvol_{wchg}$	
	Coef.	t-stat	Coef.	t-stat	Coef.	t-stat	Coef.	t-stat
$lRC_{wchg,30m}$	$-$**0.0150**	$-$**0.15**	**0**.**0357**	**0.35**	$-$**0**.**0039**	$-$**0.04**	**0.0088**	**1.06**
$lRC_{wchg,1d}$	**0.0893**	**0.54**	**0.0547**	**0.37**	**0.0795**	**0.57**	$-$0.0230**	$-$**2.57**
$DepVar_{lagged,30m}$	$-$0.1362	$-$1.02	$-$0.0651	$-$0.48	0.0787	0.44	0.0466*	1.65
$DepVar_{lagged,1d}$	$-$0.7537*	$-$1.84	0.2707	0.94	0.7112	1.50	0.1235**	2.52
$lTO_{wchg,30m}$	0.1613*	1.83	0.1189	1.39	0.0852	0.99	$-$0.0160*	$-$1.73
$lTO_{wchg,1d}$	$-$0.0103	$-$0.06	$-$0.0134	$-$0.08	$-$0.0354	$-$0.23	0.0249**	1.97
$|R|_{30m}$	0.5076	1.29	0.5829	1.52	0.6939*	1.87	0.0036	0.66
$|R|_{1d}$	$-$0.4168	$-$0.34	$-$0.3281	$-$0.28	$-$0.4496	$-$0.40	$-$0.0437	$-$1.28
$R_{30m}$	0.0726	0.15	$-$0.2457	$-$0.46	$-$0.7763	$-$1.18	0.0173***	2.68
$R_{1d}$	0.4446	1.55	$-$0.5941	$-$1.33	$-$0.8711	$-$1.35	$-$0.0091	$-$1.17
$IVOL_{30m}$	0.0940	0.24	$-$0.0123	$-$0.03	$-$0.0107	$-$0.03	$-$0.0004	$-$0.04
$IVOL_{1d}$	0.1712	0.20	0.0963	0.12	0.2023	0.26	0.0351	1.26
*Intercept*	0.8709*	1.76	0.7277	1.50	0.8074*	1.68	$-$0.0313	$-$0.62
Time FE & Trend	*yes*		*yes*		*yes*		*yes*	
Adj. $R^{2}$	0.04		0.04		0.04		0.01	
Obs	3332		3332		3332		3310	

*$$p<0.1$$, **$$p<0.05$$, ***$$p<0.01$$

This table reports the regression estimates for the effect of Reddit comments on GameStop excess returns and marketable order imbalances. The dependent variables are: i) $AR(\beta _{ges})$, the abnormal return calculated with the market model and $\beta$ for the whole sample period; ii) $AR(\beta _{30})$, same as i) but calculated with a rolling beta over 30 days; iii) $AR(\beta _{7})$, same as i) but calculated with a rolling beta over 7 days; and iv) *mroibvol*, the turnover of marketable retail order imbalance as introduced by Boehmer et al. (2021). The independent variables of interest are: i) $lRC_{30m}$ which denotes the log number of Reddit Comments on GameStop during the preceding 30-minute window and ii) $lRC_{1d}$, which is defined in the same way as i) but counts all comments made on the previous day instead of the preceding 30-minute window. For *mroibvol* and the independent variables of interest we employ weekly differences (*wchg*) to achieve stationarity and avoid trend and seasonality bias. Additionally, the following control variables are used: i) *DepVar*, a lagged measure of the dependent variable for the preceding 30-minute window and the previous day; ii)*lTO* the log of share turnover; iii) |*R*|, the absolute return of GameStop shares; iv) *R*, the return of GameStop shares and v) *IVOL*, the idiosyncratic volatility. All independent variables are measured at least one period ahead of the dependent variable and are standardized with zero mean and a standard deviation of one to make coefficients comparable. Additionally, we add Time Fixed Effects (for all 30-minute windows and weekdays) & a trend variable (counting months from start of sample). We use the Newey and West (1987) methodology with 13 lags to correct standard errors for potential heteroskedasticity and autocorrelation. The sample period is January 2020–March 2021; independent variables of interest are shown in bold

In Table 8, results of the regression of the weekly difference in log comments on abnormal returns for the following 30-min window are displayed. While the first column is calculated using a whole-sample beta, columns two and three use the 30-day rolling beta, respectively, the 7-day rolling beta. We find no significant effect of the weekly difference in Reddit comments on abnormal returns for all three variations. control variables are also overwhelmingly insignificant, indicating that abnormal returns can’t be predicted with available variables.

In column four, we use the marketable retail order imbalance (*mroibvol*) as dependent variable. Boehmer et al. (2021) showed that stocks with a positive retail order imbalance outperformed other stocks over one-week horizons and thus a positive relationship could enable us to establish another (indirect) link between Reddit comments and abnormal returns. However, as for abnormal returns, we do not find a significant relationship between Reddit comments and trading activity and almost all coefficients in this model remain small and negative.

Neither the change in the number of comments, nor the remaining control variables seem to have a significant impact on retail order imbalance or directly on abnormal returns. The low explanatory value of this model is also evidenced by the very small R-squared ratios of 0.01 for *mroibvol* and 0.04 for the excess return variables. This result is consistent with multiple prior studies (e.g., Antweiler and Frank 2004; Ammann and Schaub 2021, and others) which also demonstrated an impact of online activity and attention on trading volume but found that this online activity and the resulting investor attention is not informative.

Thus, investors are not able to exploit the relationship between online activity and trading volume in the following 30-minute window and cannot achieve abnormal returns over that time horizon. While one common explanation for this is simply that Reddit users posting comments lack information and do not add value, we cannot rule out that a nonlinear and complex interdependence between Reddit comments and trading activity or even reverse causality plays a role and prevents a measurable effect. Additionally, we also cannot rule out that informativeness of Reddit comment for abnormal returns can be found over longer time horizons.

### Results of the Granger causality test

As an additional robustness check and due to prevalent autocorrelation and results of bi-directional effects in previous studies, we perform a Granger Causality Test for the effect of weekly changes of Reddit comments on trading volume and vice versa, as well as for the relationship between Reddit comments and the retail trading proportion. We choose 13 lags for the test as a full trading day consists of 13 half-hour windows.

**Table 9:** Granger Causality test - trading volume

Trading Volume				
$H_{0}$	$lRC_{wchg}$ $\nrightarrow$ $\, Volume_{wchg}$		$Volume_{wchg}$ $\nrightarrow$ $\, lRC_{wchg}$	
	F-stat	*p*-value	F-stat	*p*-value
$lVolume_{wchg}$	4.8302***	0.0000	7.4546***	0.0000
$lTO_{wchg}$	4.3511***	0.0000	9.3595***	0.0000
$lOTO_{wchg}$	4.5863***	0.0000	5.1709***	0.0000
$lTRD_{wchg}$	2.8905***	0.0004	9.5083***	0.0000

*$$p<0.1$$, **$$p<0.05$$, ***$$p<0.01$$

This table shows the results of the Granger causality test for trading volume measures. $lRC_{wchg,30m}$ denotes the difference between the log number of all Reddit Comments on GameStop during a 30-min window and the same measure for the preceding week. The tested trading volume measures, which are also differenced with a one-week lag, are: i) *lVolume*, the logarithm of number of shares traded; ii) *lTO*, the log share turnover; iii) *lOTO*, the log option turnover and iv) *lTRD*, the log number of share trades. $H_{0}$ for the first 2 columns is that $lRC_{wchg,30m}$ does not Granger cause the trading volume change and the opposite for the last 2 columns. The sample period is January 2020–March 2021; as one trading day consists of 13 30-minute periods, 13 lags are used for the test

Results for the first test can be found in Table 9. It can be seen that the null hypothesis

$$\begin{aligned} lRC_{wchg} \nrightarrow \, Volume_{wchg} \end{aligned}$$

can be discarded with significance at the 1% level for all specifications, confirming our results of a significant impact of Reddit comments on future GameStop trading volume. However, the mirrored relationship is also significant for all measures with even higher F-statistics. While we did not systematically test for reverse causality with a lagged and controlled model, it seems likely that there is also an effect of trading volume on future Reddit comments, as has also been demonstrated for e.g., Yahoo! Finance comments (see Antweiler and Frank 2004) or Twitter posts (see Behrendt and Schmidt 2018) and others. In this line of thinking, an increase in trading volume would cause investors to divert attention to the stock and discuss it in social media. Whether this effect is completely independent of the effect in the opposite direction that we demonstrated above for WallStreetBets comments and GameStop or whether there is some systematic interaction between both effects remains to be shown in a larger, cross-sectional study.

**Table 10:** Granger causality test - retail trading proportion

Retail Trading Proportion				
$H_{0}$	$lRC_{wchg}$ $\nrightarrow$ $\, RTP_{wchg}$		$RTP_{wchg}$ $\nrightarrow$ $\, lRC_{wchg}$	
	F-stat	p-value	F-stat	p-value
$RTP(OL)_{wchg}$	1.5831*	0.0823	1.7177*	0.0510
$RTP(ST)_{wchg}$	3.9110***	0.0000	2.2296***	0.0067
$RTP(OC)_{wchg}$	1.2464	0.2390	0.9394	0.5105
$RTP(MR)_{wchg}$	2.7704***	0.0006	1.0333	0.4154

*$$p<0.1$$, **$$p<0.05$$, ***$$p<0.01$$

This table shows the results of the Granger causality test for the retail trading proportion. $lRC_{wchg,30m}$ denotes the difference between the log number of all Reddit Comments on GameStop during a 30-minute window and the same measure for the preceding week. The tested retail trading proportion measures, which are also differenced with a one-week lag, are: i) *RTP*(*OL*), the log retail share turnover measured by oddlot trades; ii) *RTP*(*ST*), the log retail share turnover measured by small trades below USD 5,000; iii) *RTP*(*OC*), the log retail option turnover measured by one-contract option trades and iv) *RTP*(*MR*), the log retail share turnover, measured by marketable retail orders. $H_{0}$ for the first 2 columns is that $lRC_{wchg,30m}$ does not Granger cause the retail trading proportion change and the opposite for the last 2 columns. The sample period is January 2020–March 2021; as one trading day consists of 13.30-min periods, 13 lags are used for the test

Granger causality for retail trading proportions can be found in Table 10. While the null hypothesis of no significant impact of Reddit comments on the weekly change of retail trading proportion can only be discarded with high confidence for the small-trades and marketable-retail-based variables, this was already expected due to the non-significant results in Table 7. However, with the exception of the small-trades-based measure and in accordance to our interpretation of the results for trading volume, all F-statistics for the opposite effect of retail trading proportion on comments are lower and non-significant. While not very convincing on itself, this result adds a further small piece of evidence to our assumption of a causal impact of Reddit comments on future trading volume, regardless of a possible bi-directional effect.

## Conclusion

In this article, we use the unique situation that arose around the GameStop share during 2020 and 2021 with highly elevated trading and investor attention to establish a link between social media activity and the trading volume in shares and options of a company.

Due to the unprecedented social media activity on Reddit’s WallStreetBets board with more than four million posts directly related to GameStop in our dataset, our results are not only robust over different specifications of trading volume but also significant for high-frequency intraday data. Finding a significant effect on trading volume but not on abnormal returns, our results confirm earlier studies like (e.g., Tumarkin and Whitelaw 2001; Antweiler and Frank 2004; Kim and Kim 2014; Ammann and Schaub 2021) in a recent high-impact scenario and extend the literature by documenting an impact on option turnover as well. The results are in accordance with an attention-based mechanism that drives trading volume after frequent exposure.

However, our results come with several limitations: We are not able to find a distinct effect of WallStreetBets comments on retail volume or trading proportion specifically, some results indicate a more complex and nonlinear relation over different time horizons and the Granger Causality test also suggests a bi-directional effect of trading volume on Reddit comments. Thus, we cannot establish causality for our findings and alternative explanations for the volume effect are possible as well. A larger cross-sectional study, optimally augmented with broker-sourced, individual-level trade data, would be necessary to confirm our findings, prove a causal effect and disentangle the possible mechanisms in which online comments affect trading activity.

## Supplementary Information

Below is the link to the electronic supplementary material.

Supplementary file 1 (pdf 168 KB)

## Data Availability

Aggregated Reddit comment data is available from the authors upon request. Due to privacy concerns, the authors do not plan to share the full Reddit comment dataset.

## Footnotes

1 see e.g., https://markets.businessinsider.com/news/stocks/retail-investors-quarter-of-stock-market-coronavirus-volatility-trading-citadel-2020-7.

2 According to his written testimony before the US House Committee on Financial Services on May 6th, 2021, which can be found at https://financialservices.house.gov/uploadedfiles/hhrg-117-ba00-wstate-genslerg-20210506.pdf.

3 For example WSB users frequently call themselves “autists” or “apes”; an (incomplete) glossary can be found at https://www.reddit.com/r/wallstreetbets/comments/l7fr21/basic_guide_to_wallstreetbets_culture_for/.

4 However, despite this focus, Pennystocks are banned from discussion on WallStreetBets due to the prevalence of pump&dump schemes.

5 E.g., so-called Yolo or Loss Porn posts, where users post screenshots to validate their portfolio positions and/or trades, are highly encouraged and cheered upon by other users on WallStreetBets.

6 Thereof famously user u$\backslash$deep[*expletive*]value, who early on invested a large part of his portfolio in GameStop shares, posted regular updates about his investments and was cited to testify in front of a Congressional Hearing about his involvement later on.

7 See https://www.cnbc.com/2021/02/17/interactive-brokers-chairman-thomas-peterffy-on-gamestop-frenzy.html.

8 Source: https://www.cnbc.com/2021/02/13/why-retail-investors-are-here-to-stay.html.

9 According to data provider SimilarWeb.

10 See e.g., https://www.bloomberg.com/news/articles/2020-05-22/options-are-now-all-the-rage-for-bored-day-traders-locked-inside or https://www.barrons.com/articles/how-retail-investors-are-fueling-the-nasdaqs-wild-ride-51599866516.

11 E.g., if half-hour *t* is on a Monday, $t-1d$ relates to the previous Friday.

12 In unreported results for 30-minute differences and STL decomposition of the retail trading proportion, we get similar results.

## Declarations

### Code availability

Jupyter notebooks containing the Python code used for this work are available from the authors upon request. The authors plan to share their notebooks and code in a public repository.
