# Supplementary material for: How online discussion board activity affects stock trading: the case of GameStop
Source: Financ Mark Portf Mang. 2022 Mar 30;36(4):443–72. doi: 10.1007/s11408-022-00407-w (PMC8965552; doi:10.1007/s11408-022-00407-w)
Supplement: Supplementary file 1 — (pdf 168 KB) [file 11408_2022_407_MOESM1_ESM.pdf]

Supplementary Material

How online discussion board activity affects stock trading: the case of GameStop

Table 11: Effect of Reddit Comments on GameStop Retail Trading Volume - Half-Hour Differences

This table reports the regression estimates for the effect of Reddit comments on retail trading measures in 30-minute windows. The dependent variables are: i) $lRTO(OL)$, the log retail share turnover measured by oddlot trades; ii) $lRTO(ST)$, the log retail share turnover measured by small trades below USD 5,000; iii) $lROTO(OC)$, the log retail option turnover measured by one-contract option trades and iv) $lRTO(MR)$, the log retail share turnover, measured by marketable retail orders. The independent variables of interest are: i) lRC_{30m} which denotes the log number of Reddit Comments on GameStop during the preceding 30-minute window and ii) lRC_{1d}, which is defined in the same way as i) but counts all comments made on the previous day instead of the preceding 30-minute window. For both, the dependent variables and the independent variables of interest, we apply first-differences (chg) to achieve stationarity. Additionally, the following control variables are used: i) $DepVar$, a lagged measure of the dependent variable for the preceding 30-minute window and the previous day; ii)lTO the log of share turnover; iii) $|R|$, the absolute return of GameStop shares; iv) R, the return of GameStop shares and v) $IVOL$, the idiosyncratic volatility. All independent variables are measured at least one period ahead of the dependent variable and are standardized with zero mean and a standard deviation of one to make coefficients comparable. Additionally we add Time Fixed Effects (for all 30-minute windows and weekdays) & a trend variable (counting months from start of sample). We use the **?** methodology with 13 lags to correct standard errors for potential heteroskedasticity and autocorrelation. The sample period is Jan. 2020 - Mar. 2021; independent variables of interest are shown in bold.

	Retail Trading Volume, First Differences (30-minute periods)							
	$lRTO(OL)_{chg}$		$lRTO(ST)_{chg}$		$lROTO(OC)_{chg}$		$lRTO(MR)_{chg}$	
	Coef.	t-stat	Coef.	t-stat	Coef.	t-stat	Coef.	t-stat
$lRC_{chg,30m}$	**0.0180***	**1.82**	**0.0220*****	**2.80**	**0.0381*****	**3.42**	**0.0288****	**2.39**
$lRC_{chg,1d}$	**−0.0009**	**−0.13**	**−0.0011**	**−0.20**	**0.0066**	**0.65**	**0.0040**	**0.51**
$DepVar_{lagged,30m}$	−0.4625***	−15.45	−0.3661***	−10.49	−0.3841***	−19.54	−0.3951***	−18.67
$DepVar_{lagged,1d}$	−0.0751*	−1.90	−0.0375	−0.94	−0.0242	−1.33	−0.0545**	−2.35
$lTO_{chg,30m}$	0.0602***	2.87	0.0167	0.94	0.0393**	2.51	0.0429**	2.32
$lTO_{chg,1d}$	0.0305	1.23	0.0049	0.22	−0.0067	−0.60	0.0144	0.77
$\|R\|_{30m}$	0.0402***	3.99	0.0282***	2.94	0.0164	1.49	0.0437***	3.27
$\|R\|_{1d}$	0.0303	0.85	0.0293	0.95	−0.0029	−0.08	0.0193	0.55
R_{30m}	0.0438***	2.85	0.0297**	2.20	0.0432**	2.41	0.0343***	2.76
R_{1d}	0.0029	0.37	0.0034	0.50	0.0042	0.60	0.0040	0.53
$IVOL_{30m}$	−0.0610***	−4.09	−0.0479***	−3.62	−0.0344***	−2.90	−0.0545***	−4.04
$IVOL_{1d}$	−0.0081	−0.27	−0.0119	−0.45	0.0095	0.29	−0.0051	−0.17
$Intercept$	0.5694***	11.38	0.0775*	1.70	0.7896***	12.21	0.5597***	9.17
Time FE & Trend	*yes*		*yes*		*yes*		*yes*	
Adj. R^2	0.34		0.36		0.23		0.23	
Obs	3554		3554		3551		3554	

$^{*}p < 0.1$, $^{**}p < 0.05$, $^{***}p < 0.01$

Table 12: Effect of Reddit Comments on GameStop Retail Trading Volume - Seasonal-Trend Decomposition

This table reports the regression estimates for the effect of Reddit comments on retail trading measures in 30-minute windows. The dependent variables are: i) $lRTO(OL)$, the log retail share turnover measured by oddlot trades; ii) $lRTO(ST)$, the log retail share turnover measured by small trades below USD 5,000; iii) $lROTO(OC)$, the log retail option turnover measured by one-contract option trades and iv) $lRTO(MR)$, the log retail share turnover, measured by marketable retail orders. The independent variables of interest are: i) lRC_{30m} which denotes the log number of Reddit Comments on GameStop during the preceding 30-minute window and ii) lRC_{1d}, which is defined in the same way as i) but counts all comments made on the previous day instead of the preceding 30-minute window. For both, the dependent variables and the independent variables of interest, we apply seasonal-trend decomposition based on LOESS (STL) to remove trend and seasonality components. Additionally, the following control variables are used: i) $DepVar$, a lagged measure of the dependent variable for the preceding 30-minute window and the previous day; ii)lTO the log of share turnover; iii) $|R|$, the absolute return of GameStop shares; iv) R, the return of GameStop shares and v) $IVOL$, the idiosyncratic volatility. All independent variables are measured at least one period ahead of the dependent variable and are standardized with zero mean and a standard deviation of one to make coefficients comparable. Additionally we add Time Fixed Effects (for all 30-minute windows and weekdays) & a trend variable (counting months from start of sample). We use the **?** methodology with 13 lags to correct standard errors for potential heteroskedasticity and autocorrelation. The sample period is Jan. 2020 - Mar. 2021; independent variables of interest are shown in bold.

Retail Trading Volume, Seasonal-Trend Decomposition								
	$lRTO(OL)_{STL}$		$lRTO(ST)_{STL}$		$lROTO(OC)_{STL}$		$lRTO(MR)_{STL}$	
	Coef.	t-stat	Coef.	t-stat	Coef.	t-stat	Coef.	t-stat
$lRC_{STL,30m}$	**0.0006**	**1.16**	**0.0009****	**2.38**	**0.0025****	**2.11**	**0.0016****	**2.49**
$lRC_{STL,1d}$	**−0.0008**	**−1.60**	**−0.0006****	**−2.03**	**0.0000**	**−0.05**	**−0.0001**	**−0.09**
$DepVar_{lagged,30m}$	0.0248	0.72	0.2837***	6.37	0.2034***	4.62	0.1253***	4.35
$DepVar_{lagged,1d}$	−0.0342	−0.31	−0.1011	−1.04	−0.0028	−0.03	−0.1992***	−2.80
$lTO_{STL,30m}$	0.0051***	4.09	−0.0008	−0.82	0.0050***	2.66	0.0039***	3.57
$lTO_{STL,1d}$	−0.0012	−1.05	−0.0002	−0.26	−0.0037**	−2.00	−0.0004	−0.43
$\|R\|_{30m}$	0.0030***	3.15	0.0022***	3.24	0.0033***	2.85	0.0026***	2.60
$\|R\|_{1d}$	−0.0032	−1.28	−0.0039**	−2.08	0.0016	0.27	−0.0004	−0.12
R_{30m}	0.0013*	1.85	0.0010*	1.73	0.0024**	2.51	0.0011*	1.67
R_{1d}	−0.0005	−1.11	−0.0007*	−1.85	−0.0004	−0.48	−0.0007	−1.48
$IVOL_{30m}$	0.0003	0.30	0.0004	0.46	−0.0015	−1.01	0.0004	0.34
$IVOL_{1d}$	0.0021	1.06	0.0027*	1.90	−0.0012	−0.24	−0.0004	−0.14
Intercept	0.0026	0.82	0.0019	0.94	0.0076	0.70	−0.0014	−0.39
Time FE & Trend	*yes*		*yes*		*yes*		*yes*	
Adj. R^2	0.05		0.10		0.08		0.07	
Obs	3600		3600		3598		3600	

* $p < 0.1$, ** $p < 0.05$, *** $p < 0.01$
